# Evaluation of an anti-stigma intervention for Mexican psychiatric trainees

**DOI:** 10.1186/s40814-021-00958-1

**Published:** 2022-01-14

**Authors:** Emmeline Lagunes-Cordoba, Ruth Alcala-Lozano, Roberto Lagunes-Cordoba, Ana Fresan-Orellana, Manuela Jarrett, Jorge Gonzalez-Olvera, Graham Thornicroft, Claire Henderson

**Affiliations:** 1 Health Service and Population Research Department, King’s College London, Institute of Psychiatry, Psychology and Neuroscience, De Crespigny Park, London, SE5 8AF UK; 2 Laboratorio de Neuromodulación, Subdirección de Investigaciones Clínicas, Instituto Nacional de Psiquiatría “Ramón de la Fuente Muñíz”, Mexico City, Mexico; 3 Instituto de Investigaciones Psicológicas, Universidad Veracruzana, Xalapa, Veracruz Mexico; 4 School of Health Science at City, University of London, London, UK; 5 Comisión Nacional para la Prevención de Adicciones, Mexico City, Mexico; 6 Centre for Global Mental Health and Centre for Implementation Science, King’s College London, Institute of Psychiatry, Psychology and Neuroscience, London, UK; 7 Centre for Implementation Science, King’s College London, Institute of Psychiatry, Psychology and Neuroscience, London, UK

**Keywords:** Stigma, Intervention, Psychiatrists, Implementation, Evaluation, Discrimination

## Abstract

**Background:**

There is research evidence regarding the presence of stigmatising attitudes in psychiatrists towards people with mental illness, but a lack of studies and interventions focused on this issue in low and middle-income countries.

**Aims:**

To assess the feasibility of implementing an anti-stigma intervention for Mexican psychiatric trainees, and its potential effects.

**Methods:**

This study comprised a pre-post design with outcome measures compared between baseline and 3-month follow-up. Quantitative outcome measures were used to evaluate the potential effects of the intervention, whilst the process evaluation required the collection and analysis of both quantitative and qualitative data.

**Results:**

Twenty-nine trainees (25% of those invited) participated in the intervention, of whom 18 also participated in the follow-up assessment. Outcome measures showed the intervention had moderately large effects on reducing stereotypes and the influence of other co-workers on trainees’ own attitudes. The main mechanisms of impact identified were recognition of negative attitudes in oneself and colleagues, self-reflection about the impact of stigma, one’s own negative attitudes and recognition of one’s ability to make change. Participants accepted and were satisfied with the intervention, which many considered should be part of their routine training. However, trainees’ work overload and lack of support from the host organisation were identified as barriers to implement the intervention.

**Conclusions:**

A brief anti-stigma intervention for Mexican psychiatric trainees is feasible, potentially effective, well accepted and was considered necessary by participants. This study also suggests mechanisms of impact and mediators should be considered for developing further interventions, contributing to reducing the damaging effects that mental health-related stigma has on people’s lives.

## Key messages regarding feasibility

- What uncertainties existed regarding the feasibility?

To our knowledge, no interventions have been implemented to target stigmatising attitudes of psychiatric trainees towards people with mental health problems in Mexico. Therefore, we were uncertain if it was feasible not only to design and implement an anti-stigma intervention for this population but also to recruit and retain participants through the study, including for a post assessment. We were also uncertain whether the intervention, its content and design, would be well received and practical to be implemented in the future.

- What are the key feasibility findings?

The intervention was well received, with overall positive comments regarding its content and its suitability for the target population. Even though recruitment rates were low, 25% of the total population, participants reported there were colleagues interested in joining the intervention but could not do it due to clashes with clinical duties, and most participants reported this should be mandatory for all trainees and hospital staff. Results also suggested the intervention was potentially effective, as according to attitude questionnaires responses, participants’ attitudes improved at our 3-month follow-up.

- What are the implications of the feasibility findings for the design of the main study?

When planning for a larger study, we recommend more involvement of directors and managers, to allow interested trainees to participate and increase recruitment rates. The positive repose to the intervention, suggested this is likely to be welcome by trainees in other hospitals, so we consider a larger study be extended to other sites to also allow for comparison between this and other settings.

## Introduction

Despite their professional training, psychiatrists have been identified as a source of stigma towards the patients they treat. Service users have reported feeling stigmatised by these very professionals, manifested by a lack of interest in their personal history; diagnoses given with a negative prognosis and without empathy; involuntary disclosure due to medication side effects; lack of information about their diagnoses or treatments; being treated as children and being excluded from important decisions [1–4]. Studies have also found evidence of stigmatising attitudes in mental health professionals, who paradoxically report more pessimistic views about recovery than does the general population [5–11]. This might be explained by a phenomenon known as ‘physician’s bias’, caused by their lack of contact with fully recovered patients [3, 9]. These studies have shown that regardless of their greater knowledge, psychiatrists do not have less stereotype endorsement or more desire to interact with people with mental illness than members of the general population.

There have been some efforts to reduce stigma in mental health professionals, mainly towards people with borderline personality and substance abuse disorders [12, 13], who are commonly considered as difficult, dangerous, manipulative and poorly motivated to commit to treatment. However, there is a need for studies of anti-stigma interventions focused on other diagnoses, and that assess their feasibility, as targeting these professionals might represent a challenge due to their busy calendars and education priorities [14].

Although some researchers have developed and successfully delivered anti-stigma programmes or interventions for these professionals [15, 16], these studies have lacked evidence about their acceptability, or the mechanisms of impact involved in their potential effectiveness.

In addition to the lack of interventions tailored for mental health professionals, there is also a gap in the evidence about how such interventions can be locally customised to low- and middle-income countries [17], the categories in which most Latin American countries fall. In a recent Latin American review on the impact of stigma, researchers suggested that it is necessary to develop customised interventions for this population [18]. To our knowledge, in Mexico, a middle-income country, with relatively high prevalence of mental disorders [19], no intervention has been developed to target negative attitudes in mental health professionals.

The main aims of this study were to determine the feasibility and potential effectiveness of an anti-stigma intervention designed for Mexican psychiatric trainees.

## Methods

### Design

This study used a pre-post design with outcome measures collected at baseline and 3 months after the intervention took place. The study included collection and analysis of both quantitative and qualitative data.

### Setting

The study took place in a psychiatric hospital in Mexico City, which provides psychiatric treatment to people aged over 18 years with any mental disorder. Data collection took place between March and July 2018. The study was approved by the Psychiatry, Midwifery and Nursing Research Ethics Committee of King’s College London (HR-16/17-3957) and from the Research Ethics Committee of the hospital where this research was conducted (CEI/C/015/2017).

### Participants

Participants were psychiatric trainees at the study site. We invited all registered trainees (110 at the time of the study) to participate. We aimed to recruit 25 to 30 subjects, which according to the National Institute of Health Research guidelines [20], are the numbers suggested for feasibility studies. Trainees were invited to participate through email circulars which included the information letter and consent form. Two dates were proposed by the education director, so the intervention could be delivered in two different sessions.

### Measures

We used three outcome measures that have been used to assess stigmatising attitudes in mental health professionals: The Mental Health Provider Stigma Inventory (MHPSI) [21]; the Mexican version of the Opinion about Mental Illness (OMI-MV) scale [22] and the questionnaire from the ‘Changing Minds’ campaign [23]. The MHPSI comprises 24 items assessing stigma within the mental health service provider-client relationship, within three different dimensions: attitudes, behaviours and co-worker influence. The OMI-MV has 34 items assessing six factors: separatism, stereotyping, restrictiveness, benevolence, pessimistic prediction and stigmatisation. Crisp’s ‘Changing Minds’ campaign questionnaire [23, 24] explores eight opinions regarding seven mental disorders: depression, panic attack, schizophrenia, dementia, eating disorder, drug addiction and alcohol addiction. We also added personality disorder to the list of disorders included in the questionnaire, as psychiatrists have been found to have negative attitudes towards people with this diagnosis. Both the MHPSI and the Changing Minds questionnaires were translated into Spanish using back-translation by both native English and Spanish bilingual speakers. We asked socio-demographic questions including gender, age and academic year.

To assess the feasibility of implementing this intervention, participants also completed a satisfaction questionnaire covering their views about the intervention, its acceptability and possible mechanisms of impact. This questionnaire included 21 multiple choice and open questions (available on demand) regarding participants’ satisfaction with the intervention’s content and structure. We also included questions focused on assessing their motivations, potential mechanism of impact, the potential effects on their intended behaviour, barriers faced to take part in the intervention, and any suggestion they had to improve this [25].

A semi-structured interview was also designed to assess feasibility, including implementation and mechanisms of impact; this consisted of questions on participants’ opinions about the quality of the speakers and the content of the intervention; their preferences regarding the elements of both sessions; the possible impact of the intervention on their own attitudes and behaviours; motives to attend the course and opinions regarding the possible implementation of this intervention in the host organisation.

### Intervention

The development of the anti-stigma intervention was based on evidence regarding the development of successful anti-stigma interventions and the results of two other studies conducted at the study site [26, 27]. The intervention consisted of two group sessions of two hours each. Further details on the full Template for the Intervention Description and Replication checklist [28], are available upon request.

Session 1 included a presentation debunking common myths and stereotypes; two videos of service users talking about their experiences with mental health-related stigma, and a brief discussion about the presentation and the videos. At the end of this session, participants were required to identify any experience of frustration trainees might have had when treating a patient during the time between the first and second session, so they could discuss this on the following session.

Session 2 included a brief discussion about any experience of frustration trainees had, or any experience of discrimination patients reported to participants; a description of the results from two studies previously conducted in the same hospital focused on identifying stigmatising attitudes in psychiatrists; a presentation focused on the impact of stigma in mental health settings; a live presentation from an expert by experience; a presentation about management of difficult patients and a final discussion about the content of this session. Both sessions were delivered by the principal investigators, ELC, who is a psychiatrists and psychotherapist, with experience conducting research in this setting.

### Procedure

Before the session started, participants completed the baseline questionnaires. At the end of the second session, participants completed the satisfaction questionnaire and were invited to participate in either a focus group or an individual interview. Outcome data were collected at 3 months follow-up, as the primary end point, via email and in person.

### Data analysis

#### Quantitative analysis

We examined demographic differences in the baseline outcomes to determine whether these should be adjusted for in the analysis. Having found none, we used paired *t* tests to establish if there were differences before and after the intervention. The overall scores of each subscales of the Mexican version of the OMI and the MPHSI scales were obtained by adding the responses of each item included in the subscales. For the ‘Changing Minds’ campaign questionnaire, the responses for each of the eight items evaluating each of the eight different psychiatric disorders considered in the questionnaire were summed to obtain an overall score for each psychiatric diagnosis. Differences were considered significant for each scale when *p* < 0.05. Because the ‘Changing Minds’ campaign questionnaire addresses different diagnoses, we did not correct for multiple testing.

Effect size was calculated using Cohen’s *d*, to establish the standardised mean difference (SMD) between two groups [29]. Effect size was considered small if the SMD was 0.30‑0.50, medium if it was 0.50‑0.80 and large if it was above 0.80. The quantitative data were analysed using the SPSS software.

#### Qualitative analysis

We used thematic analysis for the interviews, focus groups and the open-ended items from the evaluation questionnaire. A combined deductive and inductive approach was used to address the feasibility and process evaluation components whilst allowing exploration of participants’ reflections on the course and its impact [30, 31]. A total of 48 codes were grouped into different themes according to their similarities, these were later rearranged into five overarching themes, all of them with two or three subthemes. To increase validity, a second reviewer analysed 10% of all the interviews using the same analytic approach. Themes were reviewed until agreement was reached.

#### Feasibility assessment

We integrated the qualitative data with results from the satisfaction questionnaire to evaluate the following eight feasibility elements: acceptability, demand, implementation, practicality, adaptation, integration, expansion and limited-efficacy testing [25], see Table 1.

**Table 1:** Feasibility elements

Acceptability	The way the target population reacts to the intervention. Focuses on establishing whether individuals consider the intervention is appropriate for them or not. Acceptability was established by assessing satisfaction; intent to continue using the intervention; appropriateness and perceived effects.
Demand	The actual use of the intervention along with the intention of using the intervention. This was established by reporting which percentage of the total target population attended any of the two sessions. This was also used to establish recruitment and retention rates.
Implementation	This element was focused on evaluating if the execution of the intervention was successful, and on establishing the factors affecting the implementation, and the efficiency and quality of the intervention.
Practicality	The ability of participants to take part in the activities included in the intervention, which was evaluated with the evaluation questionnaire and the qualitative data.
Adaptation	Any modification or alteration made to the intervention to make it more suitable in a new context. However, this element refers to adaptations made to an already existing model that it is being tested in a new setting. Because this is a newly developed intervention, only adaptations made to the original plan were described.
Integration	Establishing if the intervention fits, and can be sustained, within the host organisation. This was assessed with the evaluation questionnaire and the qualitative data.
Expansion	The potential success of extending an already tested intervention to a different setting. However, because this is an original intervention, for this element, we only reported participants’ perceptions of fitness.
Limited-efficacy testing	As its name suggests, this element seeks to measure how effective an intervention is, even in a controlled setting, this was assessed by comparing pre- and post-outcomes questionnaires’ results.

## Results

A total of 29 psychiatric trainees (26.3%) participated, 17 males and 12 females, with a mean age of 28.2 years (SD 2.22). There were trainees from every academic year, seven (24.1%) from 1st year, nine (31%) from 2nd year, five (17.2%) from 3rd year, seven (24.1%) from 4th year and one (3.4%) from 5th year. All 29 trainees attended at least one of the two sessions delivered, but only 12 completed both sessions.

Table 2 shows the mean pre- and post-intervention (3-month follow-up) scores and SD for all outcomes. The pre-post comparison OMI-MV scale scores, showed no evidence for improvement in the attitudes of assessed trainees, only one subscale, stereotypes, showed a significant improvement with a medium effect size. The comparison of the MHPSI total scores, showed a significant reduction in the scores of those trainees at follow-up, with a medium effect size. However, only the influence of co-workers’ subscale showed a significant reduction at follow-up. The comparison of the mean pre and post scores for each diagnosis in the ‘Changing Minds’ campaign questionnaire showed that there was a significant improvement, with medium effect sizes, in the attitudes towards all, but one, of the eight psychiatric disorders assessed.

**Table 2:** Pre and post scores of outcome measures

	Mean	SD	Mean	SD	***T***	***P***	***d***	Effect
	Pre-assessment		Post-assessment					
**OMI scale**								
Total score	61.12	9.34	57.82	9.35	1.94	.071	0.35	Small
Subscales								
Separatism	16.56	4.23	15.16	3.45	2.05	.056	0.36	Small
Stereotypes	7.39	1.91	6.38	1.53	3.73	**.002**	**0.58**	Medium
Restriction	6.22	1.70	6.0	2.37	.514	.614	0.10	Small
Benevolence	10.59	2.26	10.17	2.32	.907	.387	0.18	Small
Pessimism	13.17	2.40	12.11	3.61	1.34	.197	0.34	Small
Stigmatisation	7.06	1.76	6.88	1.87	.410	.687	0.10	Small
**MHPSI**								
Total score	54.59	16.14	44.64	12.9	2.496	**.024**	**0.68**	Medium
Subscales								
Attitudes	20.22	3.82	18.50	5.43	1.390	.182	0.37	Small
Behaviours	17.18	8.58	14.70	3.70	1.318	.206	0.37	Small
Influence	17.83	7.34	13.16	6.35	3.384	**.004**	**0.68**	Medium
**Changing Minds questionnaire**								
Depression	14.06	4.45	11.52	2.92	2.501	**.024**	**0.67**	Medium
Panic attack	13.94	3.75	11.59	3.20	2.485	**.024**	**0.67**	Medium
Schizophrenia	16.53	5.49	14.52	4.22	1.892	.077	0.41	Small
Dementia	18.12	5.26	15.47	4.62	2.555	**.021**	**0.53**	Medium
Eating disorder	14.41	4.24	11.88	4.00	2.198	**.043**	**0.61**	Medium
Personality disorder	18.53	6.33	14.94	4.94	2.532	**.022**	**0.63**	Medium
Drug addiction	17.65	5.02	14.23	4.60	2.542	**.022**	**0.71**	Medium
Alcohol addiction	16.94	4.61	13.94	4.93	2.149	**.047**	**0.62**	Medium

Effect size was considered small if the standardised Cohen’s *d* was 0.30‑0.50, medium if it was 0.50‑0.80 and large if it was above 0.80

### Feasibility

#### Demand

About a quarter (26.4%) of trainees participated in this intervention, so, it was not possible to establish the actual intentions of all the trainees from the target population. However, some residents who did not take part in the study, expressed that they wanted to participate but were unable to join any session because of their duties. Therefore, it is possible that the actual demand for the intervention was higher than the reach.

Results from the evaluation questionnaire and from individual interviews suggested that trainees’ main motivation to participate in the intervention was personal interest, as they recognised learning more about the subject or learning a new skill, were the main reasons behind their decision to attend the intervention sessions. Although these results account only for trainees that participated in the intervention, when participants were questioned about what they considered were the reasons why other trainees did not participate in the study, they suggested that lack of interest was one of the main factors associated with this (Table 3).

**Table 3:** Quotes summary

Acceptability	Potential benefits	*“I have improved my understanding, as a useful tool, I no longer expressed myself with my colleagues calling patients by their disorders but by their names”*
		*“What I liked the most were the videos… they showed that it is possible to almost fully recovered, a functional recovery, which is something that we don’t usually see, at least in our training”*
	Satisfaction with the intervention	*“I think the course was good, but I think it would be better to extend it, but because our time is limited, I think it was adjusted to what we can do, with what we can work, and I like it, it inspired me lots”*
		*“I think the content was good… in general I think it was complete, the examples were very illustrative, I think the quality of the course was very good”*
		*“it was very approachable (course facilitator), I think was able to transmit that energy of wanting to make a change, and that is something important because is something that can be transmitted”*
Demand	Motivating factors	*“I did not have any idea about this subject, and I wanted to learn about it, that’s what encouraged me”*
		*“Indecisiveness maybe, boredom, lack of interest, because when things are not mandatory, and we have the option of leave early, we give priority to other things and no to our education”*
Practicality		*“The time, it was very little, and it was difficult to come, because there was not that much support for us to be allowed to come because of the schedule of our activities”*
Integration		*“Yes, yes, definitely I think this course should be mandatory… I think this is something could complement our training”*
Mediators	Recognition of own negative attitudes	*“It helped us identify how is that we stigmatise them (patients) and how us can treat them badly, and how many times we limited them because our beliefs and not that much because of their illness”*
		*“it really made us be more conscious about how we stigmatise our own patients, and helped us realise how to avoid, or how to improve this, because many times is the stigma related to mental illness what cause more damage to the patient”*
	Reflection about the impact of stigma	*“it really made us be more conscious about how we stigmatise our own patients, and helped us realise how to avoid, or how to improve this, because many times is the stigma related to mental illness what cause more damage to the patient”*
	Ability to make a change	*“To realise that there are people that have started to get interested in making a change is something that gets my attention, because what I have been usually told that things are like this, this is the way it should be and is always going to be like this. Therefore, to hear that there is someone else who thinks the opposite, is like an incentive for me”*
Unintended pathways		*“We were really interested, so interested that we are looking into developing the stigma project”*

#### Acceptability

This element corresponds to participants’ opinions towards the intervention, which is covered below (see Participants’ response).

#### Implementation

According to our fidelity checklist, every element of the proposed intervention was delivered. However, only half of the proposed elements were delivered as originally intended, the other half was partially covered or had elements missing. The elements that were not delivered as intended, were the live presentation of a mental health service user; opportunity to ask questions to the service user; disconfirmation of stereotypes by the service user and history of personal recovery by the service user. These elements were not included as planned, because they were only included in the second session, rather than in both sessions.

This intervention was originally intended to include two sessions of two hours each. However, both sessions last around 1:45 min. The first one was shorter because the live presentation by a service user was not delivered, and the second because most participants arrived late.

As few participants, who attended session 1, were not able to attend session 2, another date for session 2 was scheduled, so interested participants could complete both sessions. Although this new session was aimed to include participants from session 1 who were not able to complete the course, there were seven new participants who did not attend the first one.

The proportion of individuals that came into contact with the intervention from the target population was 26.3%, as 29 out of all the 110 trainees registered at the host institution attended at least one session. However, only 12 (10.9%) trainees completed all the elements of the intervention.

#### Practicality

Some characteristics of the host institution were considered to have influenced the intervention, as some participants considered that conflicting schedules or lack of support from their direct supervisors stopped them from attending both sessions. Although this intervention was supported by the education and clinical directors, as they agreed to reduce the activities of those trainees interested in participating, results from the questionnaires suggested that not all participants felt their activities were reduced or that they did not receive encouragement from their supervisors or direct managers to participate in the intervention (Table 3).

#### Adaptation

There were two major adaptations implemented for this intervention. The inclusion of a service user from a different psychiatric hospital, and the inclusion of an extra date to deliver the second session.

#### Integration

The results showed participant of this study considered that the content of this intervention should be included as part of their psychiatric training (Table 3).

#### Expansion

Most trainees interviewed suggested that the intervention should be implemented in the host organisation, as it could potentially help them improve their attitudes. However, some participants considered that they did not receive enough support, and some others perceived that there might be some barriers to implement this intervention. The barriers identified were a possible lack of support from authorities, lack of interest from other trainees and work overload.

#### Potential effectiveness and mechanisms of impact

All 29 participants completed the initial assessment; 21 completed the assessment at the end of the second session and 18 at follow-up. Therefore, the estimation of the effect size was calculated using only the results of those trainees that were assessed at follow-up. Fourteen trainees participated in either a focus group or an individual interview. The main scores of all the scales and subscales showed a reduction at follow-up. All the scores that showed a significant change had medium effect sizes.

In order to assess which were the mechanisms associated with any possible effect on participants, qualitative and quantitative data were integrated to assess the three different elements included in the evaluation of mechanism of impact: *participants’ response*, *mediators and unintended pathways.*

##### Participants’ response

All participants completing questionnaire reported that they were satisfied (28.6%) or very satisfied (66.7%) with the course, all found that the information provided was at least very useful and most considered that the course facilitator had expertise in the field. Over three quarters considered the intervention was better (52.4%) or much better (23.8%) than expected, only five (23.8%) considered the intervention was as expected; no trainee considered it was below their expectations. Most participants also reported the content of the intervention should be included within their routine training, and that they would recommend the course to their colleagues.

Trainees considered this intervention would lead to positive benefits for their patients, as they considered this would help them improve their empathy, reduce the use of labels and the therapeutic pessimism they have towards their patients (see Table 3).

Overall, participants responded positively to the intervention. They considered the intervention was well structured and planned, and although many commented the length of the course was adequate, they also mentioned the course should be longer, or include more sessions. Regarding course content, participants considered that the elements included in the intervention were comprehensive and included useful information. The last elements related to participants’ satisfaction with the course, was the course facilitator, who was considered to be knowledgeable, approachable and non-judgemental (Table 3).

##### Mediators

There were three main mediators identified which seemed to be related to possible changes on participants’ attitudes and behaviours: *recognition of negative attitudes*; *reflection about the impact of stigma and ability to make a change*. Moreover, these mediators seemed to have been triggered by the inclusion of the following elements in the intervention: the videos of service users; the challenge of myths/stereotypes and awareness of the results of two other studies conducted in the same hospital.

Recognition of negative attitudes, in themselves or other colleagues, and reflection about the impact of stigma seemed to be important mediators of change. As most trainees mentioned that attending the intervention help them realise the great impact stigma has on their patients, and recognise that they could be considered a source of stigma (Table 3).

A further sub-theme recognised as a possible mediator was the ability to make a change. Overall, most participants mentioned that realising they could change their attitudes or that they can contribute to change this problem, was considered an important factor for them to modify their attitudes and behaviours (Table 3).

Results from the questionnaires also support that having learnt about the attitudes psychiatric trainees have towards psychiatric patients in the host organisation (a pre-existing contextual factor) was considered one of the most influential factors linked to a possible change of attitudes from participant trainees.

##### Unintended pathways

The main unanticipated outcome recognised as consequence of this intervention was the organisation of a group of psychiatric trainees to create an anti-stigma campaign at the host institution. Two trainees that participated in the intervention, created an online anti-stigma campaign called *enlazando mentes* (connecting minds) (https://es-la.facebook.com/enlazandomentes/), which aims to reduce mental health-related stigma through education. The creation of this campaign seems to have been triggered by this intervention, as formal and informal talks with the developers suggested that, although they were already interested in creating some kind of programme to target mental health-related stigma, they were motivated to finally develop an online campaign after they both participated in this study (Table 3). Additionally, these trainees have started delivering talks focused on stigma to the newly accepted trainees, as they were interested in improving the attitudes of these residents before they started their formal psychiatric training.

## Discussion

Results from this study showed that it was feasible to deliver and evaluate an anti-stigma intervention designed specifically for a sample of Mexican psychiatric trainees, as it was not necessary to make major adaptations to the proposed intervention, and it was possible to evaluate all the elements necessary to assess potential effectiveness and mechanisms of impact. However, this study also showed there are some barriers to implement this intervention, as only a quarter of the target population was able to participate. Here, we discuss participants’ views regarding the intervention and those factors associated to its demand and potential effectiveness.

Overall, this intervention was broadly accepted by the target population. Participants’ responses were very positive as many considered the intervention was useful to improve their knowledge and attitudes towards the people they treat. Unfortunately, it was not possible to compare these results with other similar studies, as none of the other studies evaluated the acceptability or the response of participants. Similarly, of the small number of studies published about anti-stigma interventions for mental health professionals, hardly any provided information regarding fidelity, adaptation or dose. Only one study assessing the effects of training in mental health staff towards people with borderline personality disorder included the percentage of time accounted for all the activities included in the intervention [32], but failed to report the actual dose participants received from these activities.

Regarding reach and demand for this intervention, these were considered moderate as over a quarter of the whole population participated in it. However, there was an overrepresentation of males (58.6%) and of trainees from 2nd year, and years 1 and 3. Even though this might limit our capacity to generalise the findings, the demand for this intervention was similar to that reported for similar studies. One study focused on evaluating the effects of an anti-stigma intervention in psychiatrists, had a similar demand for this intervention, as the recruitment rate for that study was 22% [15]. Recruitment rates from the other studies evaluating anti-stigma interventions in similar populations were more varied, as they ranged from 14 to 77.8% [16, 32, 33]. Although the number of studies assessing demand of this type of intervention in mental health professionals is very limited, they suggested there is a moderate demand for this type of intervention in this population.

The results of this study also suggest that a tailored anti-stigma course delivered to psychiatric trainees can be effective reducing stigma towards people with mental disorders, mainly by reducing stereotypes and the influence of other co-workers on trainees’ own attitudes. One of the key elements included in the intervention was tackling of common myths and stereotypes. Hence, it is probably that the emphasis given to this element may have helped participants to reduce stereotypes, as the results from the OMI-MV suggested. Although the overall scores of the MHPSI showed reduction, only the co-worker influence subscale was significant. This result could be related to the awareness trainees gained regarding the presence and impact of stigmatising attitudes in other psychiatrists, which could have been easier to identify in their colleagues rather than in themselves.

Attitudes towards specific psychiatric disorders also showed some improvement as consequence of the intervention. However, this improvement did not seem to have extended to every mental disorder, as the attitudes towards people with schizophrenia did not seem to have improved as much as for the other disorders included in the questionnaire.

Contrary to the other processes discussed above, many authors have tried to identify which are the most effective elements included in anti-stigma interventions, for them to be able to replicate or amplified these in further interventions. In our study, participants considered the inclusion of a speaker with lived experience (interpersonal contact) as one of the most important elements associated with a possible change on their attitudes. Overall, the inclusion of interpersonal contact has been considered as one of the most important elements of a successful intervention [34–37]. Other anti-stigma interventions have also included a live presentation by a person with personal experience with positive results [38–40].

The success of interpersonal contact seems to be related to three different mediators: reduction of anxiety; increment of empathy and perspective taking, and enhancement of knowledge [41]. Intergroup contact is considered an important tool to reduce, resolve or prevent conflict between groups, mainly because of its positive and strong effects of in reducing prejudice and stereotypes [42].

The results of this study also suggested that being able to identify negative attitudes in oneself was associated with a possible improvement in participants’ attitudes. Sartorius [43] and Schulze [2] have suggested that examining one’s own attitudes and increasing tolerance towards patients, are crucial steps to change mental health care professionals’ stigmatising attitudes and behaviours. Indeed, Schulze [2] suggested that before deciding to act as a de-stigmatiser, psychiatrists should be aware of the stigmatising aspects of their clinical work, as their therapeutic role could help to either reduce or increase the stigma their patients received. More recently Knaak and Patten [44] have argued that lack of awareness of their own prejudice could be considered a ‘learning need’ amongst health care providers, as they may not be aware of their own negative attitudes.

### Strengths and limitations

There are several limitations to this study. First, there were some limitations regarding sample and target population. Because the number of trainees that participated in the intervention was only 25%, and the number of participants assessed at follow-up was even smaller, it is unlikely that these results can be generalised to the whole target population. The reduced percentage of trainees that attended both sessions (10.8%) showed that despite the support of clinical leads, there were barriers that limited the reach of this intervention, including clashes with academic and clinical activities. This suggest that similar interventions might need more involvement and support from managers and clinical leads. Even though most participants seemed very positive towards the implementation of this intervention, it is possible that some trainees might have considered the intervention as irrelevant for them, which could have influenced their decision to participate. Although psychiatric training in Mexico is supposed to include a standardised curriculum, this study only targeted trainees from one specific hospital, whose actual training and experiences might have influenced their previous attitudes and responses. According to our results, trainees seemed to have been motivated to participate in this study due to previous interest in the subject or interest in learning new skills, which in addition to desirability bias, might have influence participants to respond in a positive way. Finally, the lack of a control group means that the changes identified should be interpreted only as showing potential effectiveness; however, participation on this study did lead to some behavioural changes, whilst some trainees who participated also developed their own anti-stigma campaign and introductory course, focused on mental health stigma, for new admission doctors.

The strengths of this study include the use of a framework to assess the feasibility of implementing this intervention, as it helped us identify those key elements to successfully implement this intervention, as well as to identify mechanism of impacts and other factors involved in its potential effectiveness. Similarly, to our knowledge, this is the first study conducted in Mexico trying to address negative attitudes in psychiatric trainees, who not only showed improvement of their attitudes towards people with mental illness, but who also considered this intervention, or its content, should be part of their training. This suggests a gap in current psychiatric programmes in Mexico that could be addressed with similar interventions.

## Implications

Our results showed that members from this target population are not only interested in participating in this type of interventions, but that this type of interventions could be effective in reducing stigma in these professionals. Therefore, this research supports the idea that it is feasible to implement effective anti-stigma interventions for mental health care professionals, including psychiatric trainees. Future studies could consider all the process involved in the development of the intervention, its execution and its process evaluation to improve recruitment rates, acceptability or effectiveness in these studies. Finally, other researchers or anti-stigma campaign developers can use our results to consider the mechanisms of impact and contextual factors identified in this study.

## Data Availability

The datasets generated and/or analysed during the current study are available in the King’s College London repository (https://kclpure.kcl.ac.uk/portal/en/persons/emmeline-lagunes-cordoba(e55e1eb4-130b-48f5-a56d-9d0c280ed9f8).html), but is also are available from the corresponding author on reasonable request.
